# Male gender, Charnley class C, and severity of bone defects predict the risk for aseptic loosening in the cup of ABG I hip arthroplasty

**DOI:** 10.1186/1471-2474-11-243

**Published:** 2010-10-19

**Authors:** Jiri Gallo, Vitezslav Havranek, Jana Zapletalova, Jiri Lostak

**Affiliations:** 1 Department of Orthopaedics, Palacky University Faculty of Medicine and Dentistry & Teaching Hospital, I. P. Pavlova 6, 775 20 Olomouc, Czech Republic; 2 Joint Laboratory of Optics, Palacky University, Institute of Physics, Academy of Sciences of Czech Republic, Tr. 17. listopadu 50, 772 07 Olomouc, Czech Republic; 3 Department of Biophysics, Faculty of Medicine and Dentistry, Palacky University, Hnevotinska 3, 775 15 Olomouc, Czech Republic

## Abstract

**Background:**

We studied which factor could predict aseptic loosening in ABG I hip prosthesis with hydroxyapatite coating. Aseptic loosening and periprosthetic osteolysis are believed to be caused, at least in part, by increased polyethylene (PE) wear rate via particle disease. Based on it, increased PE wear rate should be associated with aseptic loosening regardless of the type of implant.

**Methods:**

We analyzed data from 155 revisions of ABG I hip prostheses to examine the influence of patient, implant, surgery, and wear related factors on the rate of aseptic loosening at the site of the cup. This was calculated by stepwise logistic regression analysis. The stability of the implant and severity of bone defects were evaluated intraoperatively.

**Results:**

We found that men (odds ratio, OR = 5.6; *p *= 0.004), patients with Charnley class C (OR = 6.71; *p *= 0.013), those having more severe acetabular bone defects (OR = 4 for each degree of severity; *p *= 0.002), and longer time to revision surgery (OR = 1.51 for each additional year; *p *= 0.012) had a greater chance of aseptic loosening of the cup. However, aseptic loosening was not directly predicted by polyethylene wear rate in our patients.

**Conclusion:**

Severity of bone defects predicts the risk for aseptic loosening in ABG I cup. Factors potentially associated with the quality of bone bed and biomechanics of the hip might influence on the risk of aseptic loosening in this implant.

## Background

Aseptic loosening is the most frequent cause of total hip arthroplasty (THA) failure [1]. Both mechanical and biological mechanisms are potentially involved. Mechanical factors include the initially compromised fixation interface of the implant; biological factors are associated with the particle disease that expands over the initial firmly developed bone-prosthesis interface [2]. Accordingly, wear debris derived from an artificial joint triggers multiple adverse host reactions involving signaling pathways which eventually result in osteoclast-mediated bone resorption [3-5]. Based on this, periprosthetic osteolysis undermines aseptic loosening and, therefore, a higher wear rate carries a correspondingly higher risk for failure due to aseptic loosening [6].

The term "aseptic loosening" is tightly related to a finding of gross mechanical instability at the interface between implant and bone bed. The multiple factors that influence aseptic loosening can be divided into those related to the patient, the implant, or the surgery. Known influences on aseptic loosening include variables related to polyethylene wear rate, size of periprosthetic osteolysis, design-related variables (i.e. prosthetic material and shape, fixation surface, etc.), surgical experience and technique (quality of implant settlements), and primary diagnosis [7-9]. Other factors that may be important, but are not readily analyzed, include interactions between the implant and its surroundings such as composition of joint fluid, type of lubrication, individual motion/stress pattern, and genetic predisposition [10].

In our previous studies, we found that a high risk for an increased wear rate was associated with ABG I prosthesis [11]. We also found a significant association between wear rate and severity of osteolysis in ABG I prosthesis, and an unacceptably high rate of aseptic loosening in ABG I cups [12,13]. As with the latter finding, we were interested as to whether there are significant predictors of aseptic loosening in ABG I THA. The identification of such predictors may be useful in understanding the factors associated with aseptic loosening, with the goal being to prevent loosening and reduce the need for surgical revision.

## Methods

### Patients

Patients undergoing surgical revision of ABG I prosthesis between August 2000 and December 2005 were included in this study. The revised cases belonged to a group of patients who had previously undergone the operation at the author's institution between September 1994 and January 2000 (n = 506). We previously reported on the risk for high wear rate and severe bone defects in these patients [12]. The ethical committee of the institution approved the study protocol and all revisions were performed under standard conditions with the written informed consent of all study patients. A single surgeon performed the majority of revisions (>90%).

### Prosthesis

The first generation of modular, cementless hip prosthesis with hydroxyapatite coating (HAC) was used (ABG I, Howmedica, Inc., Staines, England). We described the details concerning this implant, HAC, and surgery previously [13].

### Wear measurement

The wear measurements of retrieved polyethylene liners were performed by one of the authors (VH). The measurement technique and basic characteristics are described elsewhere [11].

### Clinical and radiographic evaluation

All of the patient's hips included in the study had stable prosthesis one year after the index surgery based on a review of radiology reports. All study participants were clinically and radiographically examined prior to revision surgery using the same protocol. Anteroposterior pelvic X-rays were performed with the patient in a supine (non-weight-bearing) position. The cup position relative to the true acetabular region was determined [14], as well as the cup position in regard to the lateral part of the tear drop figure and the abduction angle of the cup. The position of the cup relative to the floor of acetabulum was graded as lateral, in contact, or medial depending on the relationship between the most medial part of the cup and Kohler's line. The abduction angle is the angle formed by a horizontal line along the teardrop, ischial tuberosities, or obturator foramina and a line along the open face of the cup. Aseptic loosening was detected intraoperatively in cases where implant instability was revealed after a weak levering of special tools for the cup/stem removal. Intraoperatively, bone defects were evaluated and distinguished at the acetabular site as no significant bone loss (type I), contained bone loss (type II), moderate uncontained bone loss (type III), severe uncontained bone loss (types IV), and pelvic discontinuity (type V) [15]. The same classification was used to evaluate bone defects in the femur.

The Charnley classification was applied to estimate the level of walking capacity, with class A indicating no disturbance in locomotion, class B indicating bilateral hip disease and normal findings in other weight-bearing joints, and class C representing severe compromise of locomotion due to multiple joint involvement [16].

### Statistics

The primary goal of the study was to identify predictors of aseptic loosening. At first, all data were analyzed using univariate analysis. The categorical variables were compared with use of Fisher's exact test. The results of continuous variables were compared with use of Mann-Whitney U test or Student's t-test depending on the result of the Shapiro-Wilk test. The accepted significance level was 0.05. Then, we chose stepwise logistic regression analysis because of the stable and unstable status of the cup. Predictors for aseptic loosening in retrieved ABG I cups were simultaneously analyzed relative to variables contained in Tables 1 and 2. A stepwise variable entry continued if the inclusion α value was less than or equal to 0.05. Variables with no significant association to the aseptic loosening of the cup were removed from the model. This sequential inclusion/exclusion of independent variables according to stepwise criteria led eventually to the selection of an independent variable that significantly influence on the variability of the dependent variable [17]. The results were interpreted as an estimate of the change in risk per unit increase of each continuous variable or difference in risk to a reference level in case of categorical variables. Statistical analysis was performed with the commercial SPSS 15.0 package (SPSS Inc., Chicago, IL, USA).

**Table 1:** Categorical variables included in the study

		Stable implant (N = 121)	Unstable cup (N = 32)		Unstable stem (N = 2)
				
Variable	Categories	N. of hips (%)	N. of hips (%)	***P***^***a***^	N. of hips (%)
Gender	Men	33 (27)	11 (34)	0.511	0 (0%)
	Women	88 (73)	21 (66)		2 (100%)

	Osteoarthritis	27 (22)	2 (6)		0 (0)
	Hip dysplasia	56 (46)	14 (44)		1 (50)
Primary diagnosis	Osteonecrosis	20 (17)	10 (31)	0.078	0 (0)
	Traumatic	9 (7)	5 (16)		0 (0)
	Inflammatory	4 (3)	1 (3)		1 (50)
	Others	5 (4)	0 (0)		0 (0)

	A	42 (35)	11 (34)		0 (0)
Charnley type	B	73 (60)	14 (44)	**0.012**	2 (100)
	C	6 (5)	7 (22)		0 (0)

Liner geometry	Neutral	96 (79)	27 (84)		2 (100)
	Hooded	25 (21)	5 (16)	0.623	0 (0)

Head material	CoCr	111 (92)	31 (97)		1 (50)
	Zirconia	10 (8)	1 (3)	0.460	1 (50)

Cup relation to KL	Laterally	10 (8)	4 (12.5)		0 (0)
	In contact	23 (19)	4 (12.5)	0.565	0 (0)
	Medially	88 (73)	24 (75)		2 (100)

True acetabular region	Yes	97 (80)	26 (81)		0 (0)
	No	24 (20)	6 (19)	1.000	2 (100)

	Type I, II	41 (34)	2 (6)		1 (50)
Acetabular BD	Type III	67 (55)	19 (60)	**0.0003**	1 (50)
	Type IV, V	13 (11)	11 (34)		0 (0)

	Type I, II	99 (82)	27 (84)		1 (50)
Femoral BD	Type III	19 (16)	5 (16)	1.000	1 (50)
	Type IV	3 (2)	0 (0)		0 (0)

	Implant removal	1 (1)	1 (3)		0 (0)
Type of surgery	Cup revision	36 (30)	8 (25)	0.489	0 (0)
	THA revision	84 (69)	23 (72)		2 (100)

^a^Fisher's exact test, KL-Kohler's line, BD-bone defects

**Table 2:** Continuous variables included in the study

Variable	Stable implant (N = 121)	Unstable cup (N = 32)	*P*
Age at surgery (yrs.)	45.6 ± 7.1	47.8 ± 5.8	0.102^a^
	45.4 (25.1-64.5)	48.3 (37.6-56.7)	

Height (cm)	165.7 ± 8.7	165.4 ± 9.5	0.864^a^
	165 (150-195)	165 (149-187)	

Weight (kg)	75.1 ± 14.3	75.2 ± 14.8	0.918^b^
	73 (42-114)	73 (50-105)	

Cup size (mm)	50.2 ± 3.7	50.5 ± 4.1	0.830^b^
	50 (46-60)	49 (46-60)	

PE liner thickness (mm)	7.0 ± 1.9	7.2 ± 2.0	0.805^b^
	6.9 (2.8-11.9)	6.4 (4.9-11.9)	

Abduction angle of the cup (°)	46.0 ± 8.2	43.0 ± 6.3	0.073^b^
	46 (28-72)	43 (28-54)	

Time to revision surgery (yrs.)	5.7 ± 1.8	6.9 ± 1.8	**0.002**^a^
	5.7 (2.0-10.4)	6.9 (3.7-10.5)	

Linear wear rate (mm/yrs.)	0.42 ± 0.38	0.39 ± 0.32	0.764^b^
	0.35 (0.0-2.28)	0.31 (0.0-1.32)	

Volumetric wear rate (mm^3^/yrs.)	157 ± 139	144 ± 119	0.774^b^
	127 (0-815)	107 (12-467)	

Mean ± SD (standard deviation), Median (Range), ^a ^Student's t-test, ^b ^Mann-Whitney U-test, PE-polyethylene

## Results

The study included 155 patients (44 men, 111 women) who had surgical revisions of THA after a mean of 6 years from the index surgery (5.96 ± 0.15 years; mean ± standard deviation, SD). The reasons for revision were periprosthetic osteolysis (n = 115; 74%), aseptic loosening of the cup (n = 32; 21%) or stem (n = 2; 1%), and periprosthetic fracture of the femur around stable stem (n = 6; 4%). Therefore, 34 implants (22%) were unstable at the time of revision surgery. Mean polyethylene linear and volumetric wear rates were 0.415 mm/y (0-2.284; SD 0.364) and 153 mm^3^/y (0-815; SD 134.4), respectively.

### Univariate analysis

Patients with stable total hip arthroplasty at the time of revision surgery and those with mechanically unstable cup differed significantly in terms of Charnley type (*p = *0.012), severity of acetabular bone defects (*p = *0.0003), and time to revision surgery (*p = *0.002). Differences in other variables were insignificant (Tables 1 and 2).

In addition, we found a significantly higher polyethylene wear rate in type III femoral bone loss in comparison to bone defects of type I and II (Mann-Whitney U test; *p *= 0.007 and *p *= 0.015 for linear and volumetric wear rate, respectively).

### Stepwise logistic regression

Among the variables (Tables 1 and 2) that were included in the logistic regression, five significantly predicted an aseptic loosening rate in the ABG I cup (Table 3). The most surprising finding was a potential role for Charnley C patient type, assuming that these patients have the lowest physical load on the hip. On the other hand, no role was detected for polyethylene wear rate. Logistic regression can determine the percent of variance in the dependent variable explained by the independents to assess the relative importance of independents. Here, the percentage of the variance in the dependent variable explained by the independent variables was above 37% (R^2 ^= 0.375) and the -2 log likelihood achieved was beneath 100 indicating a moderate fit of the regression model to the aseptic loosening data.

**Table 3:** Variables associated with the probability of aseptic loosening of the cup in ABG I prosthesis

Variable	Odds ratio	95% confidence interval		p value	**R**^**2**^
		*Lower bound*	*Upper bound*		
Men	**5.6**	1.729	18.125	**0.004**	0.375

Charnley C type of patient	**6.71**	1.498	30.047	**0.013**	

Increasing acetabular BD by 1 step of severity	**4.06**	1.647	9.980	**0.002**	

Increasing FU by 1 year	**1.51**	1.096	2.086	**0.012**	

Increasing abduction angle of the cup by 1°	**0.91**	0.846	0.987	**0.022**	

BD-bone defects; FU-follow-up; R^2^-Nagelkerke R Square

The vast majority of our patients (109 of 155; 70%) were treated by complete revision of both acetabular and femoral components despite the fact that only two of them experienced loosened stem. The decision to remove stable stem was primarily dependent on surgeon's pre-/intraoperative estimation of its risk for premature failure. In this line, we found that only linear polyethylene wear rate predicts the risk for complete revision (OR = 36.94 per each mm/year; 95% CI 4.69-290.66; R^2 ^= 0.252).

## Discussion

According to our analysis, risk for aseptic loosening of the ABG I cup was significantly higher in men, Charnley type C patients, and those having more severe acetabular bone defects and longer time to surgery. Conversely, a decreased risk for aseptic loosening of the cup was found in patients with higher abduction angle of the cup. On the other hand, the study failed to reveal a direct association between the high polyethylene wear rate and the risk for mechanical loosening of the cup found in an identical type of cementless hip prosthesis.

Aseptic loosening of total hip arthroplasty seems to be a result of a harmful combination of mechanical and biological events destroying the bond between implant and bone bed [2]. Biological mechanisms are tightly associated with increased generation of polyethylene particles, as demonstrated in previous studies [18-20]. Particles can trigger a complex biological reaction involving the development of a chronic inflammatory microenvironment, increased number/activity of osteoclasts at the bone-implant interface, and accumulation of fluid inside the artificial joint [5,21,22]. Hypothetically, these mechanisms would together or independently lead to development of bone defects eventually resulting in aseptic loosening of the implant undermined by bone defects. Because the above-mentioned pathways depend on a continuous delivery of huge amounts of particles [6] a relationship between aseptic loosening and polyethylene wear rate is expected. In fact, we failed to demonstrate a direct association between high polyethylene wear rate and aseptic loosening of the ABG I cup. On the other hand, we revealed a strong predictive power of severity of acetabular bone defects (i.e. size of osteolysis) for aseptic loosening of the ABG I cup. These findings may suggest that periprosthetic osteolysis is an essential pre-requisite for aseptic loosening that, however, requires terminal impulse such as mechanical stress/strain inducing movement of the implant.

It has been previously determined that there is an increased risk of aseptic loosening in men comparing to woman [7,23-25]. Numerous factors might be responsible for this difference, including a higher mechanical stresses at the hip, influencing both the bone-implant interface stability and polyethylene wear rate. In this line some authors analyze the success of total hip arthroplasty in terms of biomechanically favorable hip joint conditions [26]. There may be a role for differences in hip kinematics between men and women in the explanation of gender effects [27]. Recently, Flugsrud et al. observed the existence of increased risk of early revision due to a loose cup in younger men, with a high level of physical activity during leisure time [28]. Unfortunately, there were no data available to allow us to investigate deeply this association.

A question remains as to what other factors could be behind the destabilization of the implant together with the severity of bone defects. Of these, increasing length of follow-up was found to influence the probability of aseptic loosening. Time from index surgery should be related especially to the accumulation of fatigue changes that, in conjunction with increasing size of bone defects, compromise the prosthetic-bone interface leading to degradation of fixation interface with loosening of the implant. Additionally, there is ongoing concern regarding the integrity of HAC, such as the potential for debonding of hydroxyapatite layers from a substrate or dissolution of HAC, both possibly resulting in the loss of bone-implant interface and aseptic loosening [20,29]. In our experience, ABG I cup did not work well during follow-up periods even less than 10 years, with an overall 12-year cumulative survival of 0.55 (95% CI, 0.443-0.659) [13]. Several other studies including registry-based ones has confirmed our experiences with ABG I [30-33]. Catastrophic wear in THA could be prevented at least partially by utilizing alternative bearing surfaces such ceramic on ceramic, especially in younger patients.

It is not clear why patients of Charnley type C could be predisposed to aseptic loosening of the cup. Their hips should have the least exposure to repetitive and robust mechanical load in comparison to other Charnley types. However, this could be the reason as poor bone bed quality could result from the lowest periimplant bone stresses [34]. Bone homeostasis requires regular mechanical stimuli of an adequate level and time, in accordance to Wolff's law [35]. It may be assumed that patients with multilevel damage of the locomotory apparatus demonstrate lower mechanical stimuli to the bone than patients of Charnley class A or B. However, doubt has emerged recently concerning the validity of Charnley classification for evaluation of true physical level [36]. Moreover, a discrepancy has been found between patient data on physical activity obtained before surgery and his/her true activity after the surgery [37-39]. Finally, the relationship between periprosthetic bone turnover and the level of physical activity is not linear but exhibits myriad of multilevel interactions involving biological, mechanical, and prosthetic factors.

Our study and others found an association between increased abduction angle and risk of higher polyethylene wear rate, which then increases the severity of periprosthetic osteolysis [8,12,40]. In this line, it might be surprising that we found the risk for aseptic loosening of the ABG I cup to diminish with increasing abduction angle of the cup. It can be expected that an inappropriate cup position together with high load conditions could induce micromovements, overcoming the strength of the bone-implant interface weakened by periprosthetic osteolysis [41]. On the other hand, it should be stressed that in our study the difference in abduction angle between stable and unstable cups was not significant on univariate analysis.

The main weakness of our study is that only revised THAs were included in the analysis while those that were not revised did not affect the outcome of the study. This concern might be theoretically diminished by an adequate number of cases with stable THA in contrast to those with unstable cups.

## Conclusion

In summary, this study reported an unacceptably high rate of aseptic loosening of the cup in ABG I THA related predominantly to severe periprosthetic osteolysis. We did not identify a significant association between aseptic loosening and polyethylene wear, which suggests the involvement of other factors, in particular mechanical, to terminate aseptic loosening in periprosthetic osteolysis. Our data should be also interpreted in relation to the choice of bearing surface which is vital for the success of THA especially in younger patients.

## List of abbreviations

ABG I: the Anatomique Benoist Girard hip prosthesis of 1st generation; CI: confidence interval; HAC: hydroxyapatite coating; OR: odds ratio; SD: standard deviation; THA: total hip arthroplasty.

## Competing interests

The authors declare that they have no competing interests.

## Authors' contributions

JG organized the study, recruited THA patients into the study and collected clinical/radiographic details. VH carried out polyethylene wear measurement. JZ performed the statistical analysis. JL contributed to the discussion of the results. The manuscript was primarily written by JG but all authors read and approved the final version of manuscript.

## Authors' information

Associated Professor Jiri Gallo, MD, PhD is the chief of Department of Orthopaedics, Teaching Hospital, Faculty of Medicine and Dentistry, Palacky University, Czech Republic.

Vitezslav Havranek, MS is the researcher at the Joint Laboratory of Optics, Palacky University, Institute of Physics, Academy of Sciences of Czech Republic.

Jana Zapletalova, MS, PhD is the senior statistician at the Department of Biophysics, Faculty of Medicine, Palacky University, Czech Republic.

Jiri Lostak, MD is the clinical fellowship in orthopaedics affiliated at the Department of Orthopaedics, Teaching Hospital, Faculty of Medicine and Dentistry, Palacky University, Czech Republic.

## Pre-publication history

The pre-publication history for this paper can be accessed here:

http://www.biomedcentral.com/1471-2474/11/243/prepub
